# Preparation of Candesartan and Atorvastatin Nanoparticles by Solvent Evaporation ^†^

**DOI:** 10.3390/molecules171113221

**Published:** 2012-11-06

**Authors:** Eliska Vaculikova, Veronika Grunwaldova, Vladimir Kral, Jiri Dohnal, Josef Jampilek

**Affiliations:** 1 Faculty of Pharmacy, University of Veterinary and Pharmaceutical Sciences, Palackeho 1/3, 612 42 Brno, Czech Republic; 2 Nanotechnology Centre, VSB—Technical University of Ostrava, 17. listopadu 15/2172, 708 33 Ostrava, Czech Republic; 3 Institute of Chemical Technology, Faculty of Chemical Engineering, Technicka 5, 166 28 Prague 6, Czech Republic; 4 Institute of Inorganic Chemistry, Academy of Science, 250 68 Rez, Czech Republic; 5 Research Institute for Pharmacy and Biochemistry, Lidicka 1879/48, 602 00 Brno, Czech Republic

**Keywords:** candesartan cilexetil, atorvastatin, nanoparticles, solvent evaporation, excipients, dynamic light scattering

## Abstract

The solubility, absorption and distribution of a drug are involved in the basic aspects of oral bioavailability Solubility is an essential characteristic and influences the efficiency of the drug. Over the last ten years, the number of poorly soluble drugs has steadily increased. One of the progressive ways for increasing oral bioavaibility is the technique of nanoparticle preparation, which allows many drugs to thus reach the intended site of action. Candesartan cilexetil and atorvastatin, belonging to class II of the biopharmaceutical classification system, were chosen as model active pharmaceutical ingredients in this study. Forty samples were prepared either by antisolvent precipitation/solvent evaporation method or by the emulsion/solvent evaporation technique with various commonly used surface-active excipients as nanoparticle stabilizers. All samples were analyzed by means of dynamic light scattering. The particle size of the determined 36 nanoparticle samples was to 574 nm, whereas 32 samples contained nanoparticles of less than 200 nm. Relationships between solvents and excipients used and their amount are discussed. Based on the results the investigated solvent evaporation methods can be used as an effective and an affordable technique for the preparation of nanoparticles.

## 1. Introduction

For ensure the pharmacological activity of an active pharmaceutical ingredient (API), the solubility of the API in physiological liquids is required, so that the API can be available at the place of absorption. Solubility in various solvents is a characteristic property of a particular compound. The solubility of a compound in water correlates to a great extent with the solubility in physiological liquids and is the first limiting factor for good absorption and biodistribution. Contrary to these facts, over the last ten years, the number of poorly soluble drugs has steadily increased. Estimates suggest that 40% of the drugs in the pipelines have solubility problems. Literature states that about 60% of all drugs coming directly from synthesis nowadays are poorly soluble [1,2,3].

One of the progressive ways how to increase the solubility of an APIs is the preparation of drug nanoparticles. The technique of nanoparticle drug delivery allows many pharmacological agents to reach the desired site of action. APIs are either adjusted alone till nano size and administered in nanoparticle oral dosage forms or attached/incorporated into nanoparticles prepared from inert materials which serve as a universal drug delivery system. The advantages of nanotechnology are as follows: (i) increased bioavailability (quick dissolution; improved penetration through membranes); (ii) lower doses; (iii) lower toxicity; (iv) targeted biodistribution; (v) reduction of influence of food on variability; (vi) quicker development of formulations [2,4,5,6,7]. Nanoparticles of less than 200 nm are of practical importance [8,9,10,11,12,13]. A great problem is the insufficiently investigated possible toxicity of nanoparticles. The toxicity is dependent on the shape and surface properties of nanoparticles, because both can influence nanoparticle-cell interactions as well as the rate of penetration to cells. Among the various nanoparticle forms nanotubes were found to be one of the most toxic nanoparticle shapes [14,15,16,17].

A wide range of techniques have been developed for the preparation of nanomaterials. These methods are typically grouped into two categories: top-down (generally dispergation processes) [11,12,13,18,19,20,21] and bottom-up (generally precipitation processes) [11,12,13,18,22,23,24], whereas the latter is by far the most popular in the preparation of nanoparticles. In bottom-up methods, nanoparticles can be produced by crystallization/precipitation and solvent evaporation. Spray drying, evaporative precipitation into aqueous solution, microemulsions or supercritical fluid technology belong to the solvent evaporation methods. The liquid antisolvent (LAS) precipitation process is a noteworthy method that has been extensively studied. An excellent review dealing with this technique was published by Thorat *et al.* [25]. The current paper is aimed at verification of conditions of an effective and an affordable technique for the preparation of nanoparticles by solvent evaporation as was discussed recently [26].

A polar and nonpolar solvent were used in our research, therefore the exact principle of the applied solvent evaporation method is dependent on the water-based system, including or not an aqueous miscible organic solvent. The polar acetone (AC) and nonpolar dichloromethane (DCM) were chosen as the most suitable solvents for easy dissolution of the APIs, so two different possible mechanisms can be supposed for the nanoparticle synthesis. When API is dissolved in AC and then mixed with water containing a stabilizer, nanoparticles are formed spontaneously and immediately upon mixing. This method can be called antisolvent precipitation/solvent evaporation, and the procedure is in principle similar to the evaporative precipitation into aqueous solution [27,28] or the liquid antisolvent precipitation [25]. When the API is dissolved in DCM and then mixed with water containing stabilizers, an emulsion (o/w type) is formed; API is clustered by the excipient, which results in the encapsulation of the API into nano-vesicula. This combination of emulsification and solvent evaporation nanoparticle synthesis can be called emulsion/solvent evaporation [19,29]. 

**Figure 1:**
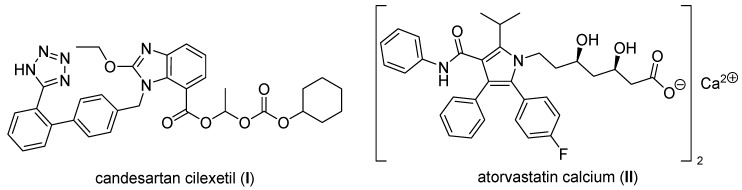
Structures of candesartan cilexetil as prodrug and atorvastatin calcium salt.

The model APIs candesartan cilexetil (**I**) and atorvastatin calcium (**II**) were chosen as representatives of poorly aqueous soluble compounds belonging to class II drugs of the biopharmaceutical classification system (BCS). Drugs of the mentioned class are characterized by low aqueous solubility and high permeability [30]. Candesartan (2-ethoxy-1-({4-[2-(2H-1,2,3,4-tetrazol-5-yl)phenyl]phenyl}methyl)-1*H*-1,3-benzodiazole-6-carboxylic acid) is an angiotensin II receptor antagonist used mainly for the treatment of hypertension. The prodrug candesartan cilexetil, see Figure 1, is marketed by AstraZeneca and Takeda Pharmaceuticals, commonly under the trade names Blopress^®^, Atacand^®^, Amias^®^, and Ratacand^®^. The use of a prodrug form increases the bioavailability of candesartan. Despite this, its absolute oral bioavailability is relatively poor (approx. 15%) [31,32]. Atorvastatin [(3*R*,5*R*)-7-[2-(4-fluorophenyl)-3-phenyl-4-(phenylcarbamoyl)-5-propan-2-ylpyrrol-1-yl]-3,5-dihydroxyheptanoic acid] inhibits HMG-CoA reductase and thus causes a decrease of cholesterol in the body. Atorvastatin is used as a calcium salt, see Figure 1, and is marketed by Pfizer under the trade names Lipitor^®^ or Sortis^®^. The low plasma concentration (approx. 12%) of atorvastatin is especially caused by an extensive first-pass metabolism in the liver, nevertheless the overall solubility of atorvastatin is strictly pH-dependent (many atorvastatin solid dosage forms are buffered, e.g., by carbonates), and administration with food produces a 25% reduction of atorvastatin absorption [33,34]. 

As mentioned, both APIs are BCS class II drugs, hence their oral bioavailability is solubility rate limited [30,31,32,35,36,37]. For enhancement of solubility of candesartan cilexetil various approaches can be used, such as pectin complexes [38], self-emulsifying drug delivery systems [39] or development of nanoparticle formulations [7,40]. The solubility of atorvastatin calcium can be enhanced, for example, using an amorphous API [41], by application of the liquisolid technique [42], formulation of self-microemulsifying drug delivery systems [43], utilization of drug-polymer interactions found due to physical mixing [44] or preparation of amorphous nanoparticles [45].

Various types of surface-active excipients were used as nanoparticle stabilizers and relationships between a substance, a solvent and a used excipient are discussed. Used excipients represent various classes of pharmaceutical adjutants (emulsifiers/viscosity modifiers/thickeners, nonionic or anionic surfactants) that can be utilized as solubility modifying compounds/nanoparticle stabilizers, such as Tween 80 (TW), sodium dodecyl sulfate (SDS), macrogol 6000 (PEG), sodium carboxymethyl cellulose (SCMC) and sodium salt of carboxymethyl dextran (SCMD). The main criteria for excipient selection were its pharmaceutical safety (all excipients are GRAS, Generally Recognized as Safe, substances) and their affordability. Based on a previous study 5% and 10% concentrations of each excipient were chosen [26]. The optimal concentration of surfactant is important for optimal particles wetting. If the concentration is too low, particles float on the surface. If the concentration is too high bubbles appear [46].

## 2. Results and Discussion

**Figure 2:**
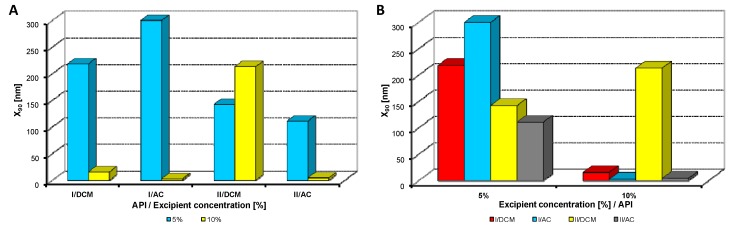
Dependence of particle size (x_90_ [nm]) of model APIs **I**, **II** on concentration [%] of Tween 80 in dichloromethane (DCM) or acetone (AC). (**A**) Samples are grouped according to APIs; (**B**) samples are grouped according to excipient percentage. For clarity sake, the values on y-axis are only to 300 nm.

**Figure 3:**
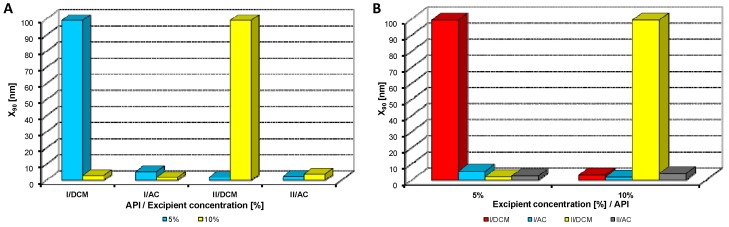
Dependence of particle size (x_90_ [nm]) of model APIs **I**, **II** on concentration [%] of sodium dodecyl sulfate in dichloromethane (DCM) or acetone (AC). (**A**) Samples are grouped according to APIs; (**B**) samples are grouped according to excipient percentage. For clarity sake, the values on y-axis are only to 100 nm.

**Figure 4:**
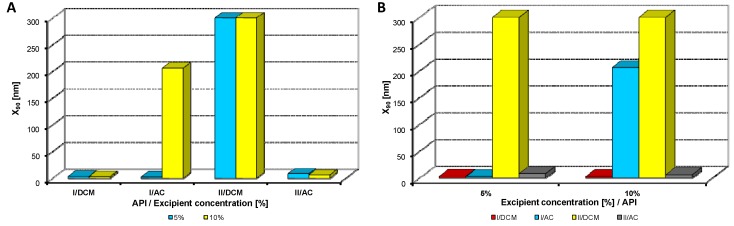
Dependence of particle size (x_90_ [nm]) of model APIs **I**, **II** on concentration [%] of macrogol 6000 in dichloromethane (DCM) or acetone (AC). (**A**) Samples are grouped according to APIs; (**B**) samples are grouped according to excipient percentage. For clarity sake, the values on y-axis are only to 300 nm.

**Figure 5:**
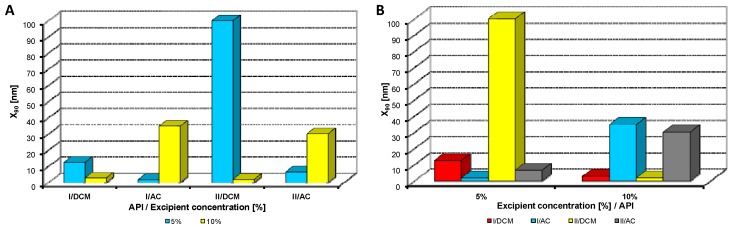
Dependence of particle size (x_90_ [nm]) of model APIs **I**, **II** on concentration [%] of sodium carboxymethyl cellulose in dichloromethane (DCM) or acetone (AC). (**A**) Samples are grouped according to APIs; (**B**) samples are grouped according to excipient percentage. For clarity sake, the values on y-axis are only to 100 nm.

**Figure 6:**
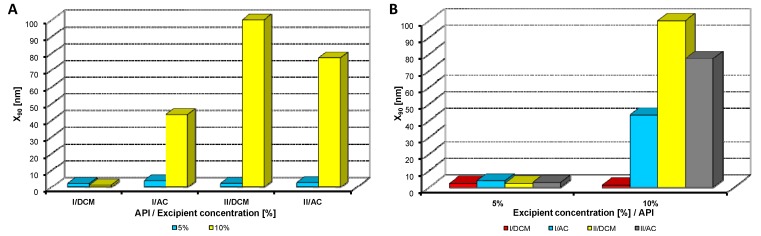
Dependence of particle size (x_90_ [nm]) of model APIs **I**, **II** on concentration [%] of sodium carboxymethyl dextran in dichloromethane (DCM) or acetone (AC). (**A**) Samples are grouped according to APIs; (**B**) samples are grouped according to excipient percentage. For clarity sake, the values on y-axis are only to 100 nm.

**Figure 7:**
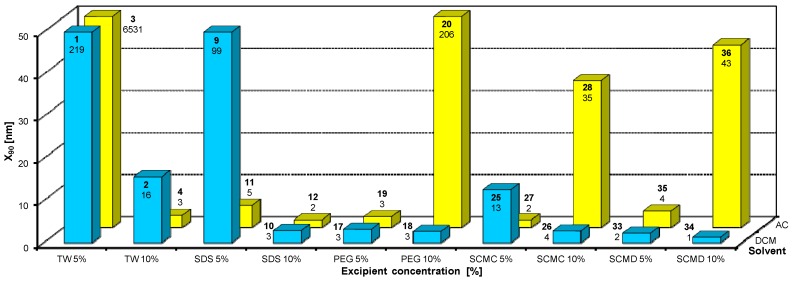
Dependence of particle size (x_90_ [nm]) of candesartan cilexetil (**I**) on concentration [%] of Tween 80 (TW), sodium dodecyl sulfate (SDS), macrogol 6000 (PEG), sodium carboxymethyl cellulose (SCMC), sodium carboxymethyl dextran (SCMD) in dichloromethane (DCM) or acetone (AC). For clarity sake, the values on y-axis are only to 50 nm.

**Figure 8:**
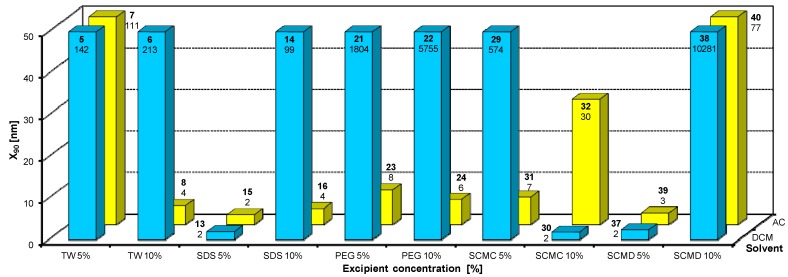
Dependence of particle size (x_90_ [nm]) of atorvastatin calcium (**II**) on concentration [%] of Tween 80 (TW), sodium dodecyl sulfate (SDS), macrogol 6000 (PEG), sodium carboxymethyl cellulose (SCMC), sodium carboxymethyl dextran (SCMD) in dichloromethane (DCM) or acetone (AC). For clarity sake, the values on y-axis are only to 50 nm.

Both model APIs **I** and **II** dissolved in dichloromethane and acetone (2% concentration) were added to aqueous solutions (5%, 10% concentration) of excipients, *i.e.*, eight samples were prepared with each excipient. The final API:excipient ratios were 1:2.5 (2%:5%), 1:5 (2%:10%). The systems were stirred for 10 min at 35 °C; afterwards the mixtures were transferred to an ultrasonic bath, where they were mixed again for 40 min, and simultaneously the organic solvent was evaporated (to final 10 mL sample volume) by self-warming of the ultrasonic bath. Then all the samples were characterized by dynamic light scattering [46]. All the results are presented in Table 1, Table 2, Table 3, Table 4, Table 5 and Figure 2, Figure 3, Figure 4, Figure 5, Figure 6, Figure 7, Figure 8. 

**Table 1:** Particle size (x_10_, x_90_ [nm]) of APIs **I**, **II** and concentration [%] of Tween 80 in dichloromethane (DCM) or acetone (AC). All the presented results are reported as the medium value of four independent measurements. The results of nano-size samples are expressed as the mean ± SD (*n* = 4 measurements). The SDs of micro-size samples are not indicated due to the measurability range of Nanophox. Samples that contained nanoparticles <200 nm are bolded; nanoparticles <10 nm are indicated by asterisk. (S.No. = sample number).

API/Solvent	Tween 80						
	S.No.	5%		S.No.	10%		
		x_10_	x_90_		x_10_	x_90_	Particle size [nm]
**I**/DCM	**1**	160 ± 4.8	219 ± 5.6	**2**	14 ± 0.4	**16** ± 0.5 *
**I**/AC	**3**	3183	6531	**4**	2 ± 0.1	**3** ± 0.1 *
**II**/DCM	**5**	97 ± 2.9	**142** ± 4.3	**6**	145 ± 4.4	213 ± 6.4
**II**/AC	**7**	101 ± 3.0	**111** ± 3.3	**8**	3 ± 0.1	**4** ± 0.1 *

**Table 2:** Particle size (x_10_, x_90_ [nm]) of APIs **I**, **II** and concentration [%] of sodium dodecyl sulfate in dichloromethane (DCM) or acetone (AC). All the presented results are reported as the medium value of four independent measurements. The results of nano-size samples are expressed as the mean ± SD (*n* = 4 measurements). The SDs of micro-size samples are not indicated due to the measurability range of Nanophox. Samples that contained nanoparticles <200 nm are bolded; nanoparticles <10 nm are indicated by asterisk. (S.No. = sample number).

API/Solvent	Sodium dodecyl sulfate						
	S.No.	5%		S.No.	10%		
		x_10_	x_90_		x_10_	x_90_	Particle size [nm]
**I**/DCM	**9**	90 ± 2.7	**99** ± 3.0	**10**	2 ± 0.1	**3** ± 0.1 *
**I**/AC	**11**	4 ± 0.1	**5** ± 0.2 *	**12**	1 ± 0.03	**2** ± 0.1 *
**II**/DCM	**13**	1 ± 0.03	**2** ± 0.1 *	**14**	90 ± 2.7	**99** ± 3.0
**II**/AC	**15**	2 ± 0.1	**2** ±0.1 *	**16**	2 ± 0.1	**4** ± 0.1 *

**Table 3:** Particle size (x_10_, x_90_ [nm]) of APIs **I**, **II** and concentration [%] of macrogol 6000 in dichloromethane (DCM) or acetone (AC). All the presented results are reported as the medium value of four independent measurements. The results of nano-size samples are expressed as the mean ± SD (*n* = 4 measurements). The SDs of micro-size samples are not indicated due to the measurability range of Nanophox. Samples that contained nanoparticles <200 nm are bolded; nanoparticles <10 nm are indicated by asterisk. (S.No. = sample number).

API/Solvent	Macrogol 6000						
	S.No.	5%		S.No.	10%		
		x_10_	x_90_		x_10_	x_90_	Particle size [nm]
**I**/DCM	**17**	2 ± 0.1	**3** ± 0.1 *	**18**	2 ± 0.1	**3** ± 0.1 *
**I**/AC	**19**	2 ± 0.1	**3** ± 0.1 *	**20**	156 ± 4.7	206 ± 6.2
**II**/DCM	**21**	1639	1804	**22**	5231	5755
**II**/AC	**23**	6 ± 0.2	**8** ± 0.2 *	**24**	4 ± 0.1	**6** ± 0.2 *

**Table 4:** Particle size (x_10_, x_90_ [nm]) of APIs **I**, **II** and concentration [%] of sodium carboxymethyl cellulose in dichloromethane (DCM) or acetone (AC). All the presented results are reported as the medium value of four independent measurements. The results of nano-size samples are expressed as the mean ± SD (*n* = 4 measurements). The SDs of micro-size samples are not indicated due to the measurability range of Nanophox. Samples that contained nanoparticles <200 nm are bolded; nanoparticles <10 nm are indicated by asterisk. (S.No. = sample number).

**API/Solvent**	**Sodium carboxymethyl cellulose**						
	**S.No.**	**5%**		**S.No.**	**10%**		
		**x_10_**	**x_90_**		**x_10_**	**x_90_**	**Particle size [nm]**
**I**/DCM	**25**	11 ± 0.3	**13** ± 0.4	**26**	2 ± 0.1	**3** ± 0.1 *
**I**/AC	**27**	1 ± 0.03	**2** ± 0.1 *	**28**	32 ± 1.0	**35** ± 1.1
**II**/DCM	**29**	401 ± 12	574 ± 17	**30**	1 ± 0.03	**2** ± 0.1 *
**II**/AC	**31**	6 ± 0.2	**7** ± 0.2 *	**32**	27 ± 0.8	**30** ± 0.9

**Table 5:** Particle size (x_10_, x_90_ [nm]) of APIs **I**, **II** and concentration [%] of sodium carboxymethyl dextran in dichloromethane (DCM) or acetone (AC). All the presented results are reported as the medium value of four independent measurements. The results of nano-size samples are expressed as the mean ± SD (*n* = 4 measurements). The SDs of micro-size samples are not indicated due to the measurability range of Nanophox. Samples that contained nanoparticles <200 nm are bolded; nanoparticles <10 nm are indicated by asterisk. (S.No. = sample number).

API/Solvent	Sodium carboxymethyl dextran						
	S.No.	5%		S.No.	10%		
		x_10_	x_90_		x_10_	x_90_	Particle size [nm]
**I**/DCM	**33**	2 ± 0.1	**2** ± 0.1 *	**34**	1 ± 0.03	**1** ± 0.03 *
**I**/AC	**35**	3 ± 0.1	**4** ± 0.1 *	**36**	39 ± 1.2	**43** ± 1.3
**II**/DCM	**37**	2 ± 0.1	**2** ± 0.1 *	**38**	9345	10281
**II**/AC	**39**	2 ± 0.1	**3** ± 0.1 *	**40**	70 ± 2.1	**77** ± 2.3

Figure 2, Figure 3, Figure 4, Figure 5, Figure 6 illustrate the dependence of particle size expressed as the cumulative distribution x_90_ [nm] of the APIs **I**, **II** on the concentration [%] of an individual excipient, whereas in Figures A samples are grouped according to individual APIs **I**, **II**, while in Figures B individual APIs are always separated according to the percentage of the excipient. The particle size x_90_ was used for evaluation of the method success, since this value represents 90% of the cumulative particle size distribution in the measured sample. The dispersity is a measure/degree of the homogeneity/heterogeneity of sizes of particles in a mixture/system. It is possible to see this feature on the width of the particle-size distribution, which is described as differences between cumulative distribution x_10_ and x_90_, see Table 1, Table 2, Table 3, Table 4, Table 5. According to the results, when micro-size samples (**3**, **21**, **22**, **38**) were eliminated, the average relation of the cumulative distribution x_10_/x_90_ ranged from 0.6 to 0.9. It is possible to suppose that nanoparticles are spheres, because the size in dynamic light scattering represents the hydrodynamic diameter of the particle. All samples were dispersed by ultrasonics directly before the measurement to avoid possible re-agglomeration. Stabilization of the dispersed samples was achieved by surfactants and by the constant temperature. The measuring cell was equilibrated at 25 °C, so the Brown motion of nanoparticles is influenced just by their size.

From Figure 2A, Figure 3A, Figure 4A, Figure 5A, Figure 6A it can be stated that generally particle size is not dependent on the type of model API, but it is partially influenced by the type and concentration of the excipient utilized. Nevertheless, it can be supposed that in the case of candesartan cilexetil (**I**) smaller particles were found, especially when atorvastatin calcium (**II**) and SDS, PEG and SCMC in dichloromethane were used, as it is illustrated in Figure 7 and Figure 8, where the dependences of the particle size of individual APIs **I** and **II** on the concentrations of individual excipients are shown. This fact is probably caused by the solvent used, because dichloromethane has less advantageous properties in comparison with acetone, as discussed below.

Table 6 summarizes results of all the samples of nanoparticles under 900 nm size depending on solvents and the type and amount of excipients. As the aim of this contribution is specification of suitable conditions for nanoparticles preparation, in Table 6 generated nanoparticles are not divided according to used APIs.

**Table 6:** View of formed samples of nanoparticles (≤900 nm) depending on solvents and type and amount of excipients. (conc. = concentration; excp. = excipient; dichloromethane = DCM; acetone = AC; Tween 80 = TW; sodium dodecyl sulfate = SDS; macrogol 6000 = PEG; sodium carboxymethyl cellulose = SCMC; sodium carboxymethyl dextran = SCMD).

Excp. conc./type	DCM		Sum total	Overall averagex_90_ [nm]	AC		Sum total	Overall averagex_90_ [nm]
	5%	10%			5%	10%		
	number of nanop. samples				number of nanop. samples			
TW	2	2	4	147	1	2	3	39
SDS	2	2	4	51	2	2	4	3
PEG	1	1	2	3	2	2	4	56
SCMC	2	2	4	148	2	2	4	18
SCMD	2	1	3	2	2	2	4	32
**Sum total**	9	8	17	351	9	10	19	148
**Overall average** **x_90_ [nm]**	117	42	160	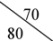	16	41	57	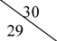

After summation of all the results it can be concluded that from 40 prepared mixtures 36 samples contained nanoparticles (see Table 1, Table 2, Table 3, Table 4, Table 5), from which 32 samples contained nanoparticles smaller than 200 nm (see Table 1, Table 2, Table 3, Table 4, Table 5, bolded values). Nanoparticles under 10 nm were determined in 22 samples from 32, see Table 1, Table 2, Table 3, Table 4, Table 5 (asterisked bolded values).

Based on the results listed in Table 6 and Figure 7 and Figure 8 it can be generally stated that the solvent used plays the crucial role in generation of nanoparticles. This fact was not so evident in the previous study, where only steroid-like compounds were investigated [26]. This effect of solvent was significant in the case of atorvastatin calcium (**II**), which is a salt and thus by its chemical nature absolutely different from other investigated model compounds. It depends on the used solvent, if the system is single-phase (acetone/water) or biphasic (dichloromethane/water, o/w type), thus whether nanoparticles will be formed spontaneously and immediately upon mixing or if emulsions will be generated and nanoparticles will not be formed spontaneously but after energy input, e.g., ultrasonic. As the way of preparation was the same (mixing and ultrasounding), it is evident from the results that the polar solvent acetone is preferable to nonpolar dichloromethane, *i.e.*, that antisolvent precipitation/solvent evaporation method is a more convenient/versatile way for preparation of nanoparticles than the emulsion/solvent evaporation technique. Results with APIs dissolved in acetone provided more nanoparticle samples comparable with dichloromethane (19/17), and the particle size of APIs dissolved in acetone was significantly smaller than that of APIs dissolved in dichloromethane (148/351).

From all the results (see Figure 7 and Figure 8) it is evident that the usage of Tween 80, especially at 5% concentration (ratio 1:2.5), and sodium carboxymethyl dextran, especially at 10% concentration (ratio 1:5), was the least advantageous as discussed previously [26]. In other cases both 5% and 10% concentrations of excipients provided similar results. Surprisingly, macrogol 6000 did not afford as good results as expected [26]. Sodium dodecyl sulfate and sodium carboxymethyl cellulose can be universally used as nanoparticle stabilizers both in dichloromethane and acetone.

## 3. Experimental

### 3.1. General

Both APIs were obtained from Zentiva (Prague, Czech Republic), all excipients were purchased from Sigma-Aldrich (Prague, Czech Republic). Dichloromethane was purchased from Merck (Darmstadt, Germany). Acetone was purchased from LachNer (Neratovice, Czech Republic). All compounds as well as solvents were of analytical grade. H_2_O-HPLC—Mili-Q Grade was used as a solvent of excipients. Particle sizes of all the final samples were determined using dynamic light scattering in a Sympatec Photon Cross-correlation Sensor Nanophox (Sympatec GmbH, System-Partikel-Technik, Clausthal-Zellerfeld, Germany), He-Ne laser 632.8 μm, intensity max. 10 mW. The measurement cell was equilibrated at 25 °C.

### 3.2. Synthesis

#### Standardized General Procedure for Preparation of Nanoparticles

Tween 80, sodium dodecyl sulfate (SDS), macrogol 6000 (PEG), sodium carboxymethyl cellulose (SCMC) and sodium carboxymethyl dextran (SCMD) were used as excipients. Each excipient (0.5 g or 1.0 g) was dissolved in water (10 mL), and two solutions with concentrations 5% and 10% were prepared. Candesartan cilexetil and atorvastatin calcium (0.2 g) were dissolved in dichloromethane or acetone (10 mL), *i.e.*, 2% solutions were prepared. The solutions of the substances in dichloromethane (DCM) or acetone (AC) were slowly dropped (2 mL/min) to the aqueous solutions of excipients that were stirred (600 rpm). Then the system was stirred (600 rpm) for 10 min at 35 °C, after which the mixtures were transferred to an ultrasonic bath in the fume chamber, where they were mixed again for 40 min, and simultaneously organic solvent was evaporated. The final volume of the aqueous sample was 10 mL. The particle size of nanonized substances in samples was evaluated by means of Nanophox. All samples were dispersed by ultrasonics directly before the measurement. Measurements were repeated four times. All the presented results are reported as the medium value of these four independent measurements. The results of nano-size samples are expressed as the mean ± SD (*n* = 4 measurements). Standard deviations of micro-size samples are not indicated due to the measurability range of Nanophox. All the results are summarized in Table 1, Table 2, Table 3, Table 4, Table 5 and illustrated in Figure 2, Figure 3, Figure 4, Figure 5, Figure 6, Figure 7, Figure 8.

## 4. Conclusions

Forty samples of candesartan cilexetil (**I**) and atorvastatin calcium (**II**) were prepared by solvent evaporation in media Tween 80, sodium dodecyl sulfate, macrogol 6000, sodium carboxymethyl cellulose and sodium carboxymethyl dextran. All the samples were analyzed by a Nanophox spectrometer. According to the cumulative distribution x_90_, 36 samples contained nanoparticles; 32 samples contained nanoparticles <200 nm; and 22 samples contained nanoparticles <10 nm. The used solvent played a crucial role in generation of nanoparticles. The polar solvent acetone was considerably more advantageous than nonpolar dichloromethane, *i.e.*, the antisolvent precipitation/ solvent evaporation method is a more convenient/versatile way for preparation of nanoparticles than the emulsion/solvent evaporation technique. The selected conditions are convenient for formation of nanoparticles, and the excipients used (except Tween 80) are in principal applicable as nanoparticle stabilizers. Sodium dodecyl sufate and sodium carboxymethyl cellulose at both concentrations tested, 5% and 10%, *i.e.*, API:excipient ratios of 1:2.5, 1:5, can be universally used as nanoparticle-stabilizing agents. It can be concluded that the investigated solvent evaporation method can be used as an effective and an affordable technique for the preparation of nanoparticles. After selection of a convenient non-toxic organic solvent this method can be scaled up. Nanoparticles of candesartan cilexetil or atorvastatin prepared in this manner would be subsequently used for nanoparticle formulations with supposed enhanced bioavailability.
